# HIV and Hepatitis B co-infection in Tanzania: Analysis of the 2022–2023 Tanzania HIV Impact Survey

**DOI:** 10.1371/journal.pone.0358242

**Published:** 2026-09-11

**Authors:** Felistar Mwakasungura, Alfateresia Mwasangama, Davis Amani, Elias Bukundi, Bruno Sunguya

**Affiliations:** 1 Department of Community Health, School of Public Health and Social Sciences, Muhimbili University of Health and Allied Sciences, Dar es Salaam, Tanzania; 2 Department of Epidemiology and Biostatistics, School of Public Health and Social Sciences, Muhimbili University of Health and Allied Sciences, Dar es Salaam, Tanzania; 3 School of Nursing and Midwifery, Aga Khan University, Dar es Salaam, Tanzania; 4 Department of Epidemiology and Biostatistics, School of Public Health and Family Medicine, University of Cape Town, Cape Town, South Africa; East Carolina University Brody School of Medicine, UNITED STATES OF AMERICA

## Abstract

Co-occurrence of HIV and Hepatitis B Virus (HBV) in sub-Saharan Africa, although less prevalent, is often more serious and exhibits rapid clinical deterioration even with appropriate treatment, jeopardizing efforts to reach regional and global targets. Evidence varies in the region and not reported in some countries. We used data from the nationally representative survey to characterize the burden and determinants of HIV and HBV co-occurrence in Tanzania. This secondary data analysis of the Tanzania HIV Impact Survey (THIS) involved 33,263 individuals aged 15 years and above, randomly selected from 14,966,262 households from 31 regions of Tanzania. The main outcome variable was HIV and HBV coinfections. Analyses were conducted using descriptive analysis, to estimate prevalence of co-occurrences, while Chi-square test used to characterize the burden. Through bivariable and multivariable binomial logistic regression models, we could estimate the associations between different independent variables with HIV-HBV co-occurrence. The burdens of HIV and HBV in the general population were 4.4% and 3.5% respectively. The prevalence of dual HIV and HBV infections was 0.3%. Among people diagnosed with HIV, 6.2% had HBV co-occurrence. After adjusting for confounder and other variables, age was significantly associated with coinfection (aOR = 1.02; 95% CI: 1.01–1.03; p < 0.001). Participants who were not in union had significantly higher risk of coinfection compared with those in union (aOR = 1.56; 95% CI: 1.01–2.41; p = 0.047). Having one lifetime sexual partner remained strongly protective factor against HIV-HBV coinfection (aOR = 0.19; 95% CI: 0.09–0.42; p < 0.001). Although the burden of HIV/HBV coinfection remains as low as 0.3% in the general population in Tanzania, the risk remains high among older adults and those with multiple sexual partnerships. Integrating HBV screening in all opportunities presented in the successful HIV program may help addressing the dual burden. For effectiveness, more efforts should target people with high-risk sexual behaviors and those with advanced age.

## Introduction

Transmitted through similar mechanisms, there is a growing concern over an increased burden of both Human Immunodeficiency Virus (HIV) and Hepatitis B Virus (HBV) infections globally [1]. While several effective strategies and interventions have resulted into an epidemic control of HIV in the region, a growing burden of HBV threatens the gain thus far. At the end of 2024, SSA accounted for approximately two-thirds of the 40 million people reported living with HIV globally, and over 600,000 who succumbed to HIV related deaths [2]. A similar pattern is seen in people with chronic HBV, where recent statistics estimated annual global deaths of over 800,000 from HBV-associated liver cirrhosis and cancers, the majority of which also originate from this region [3]. This signifies disparities in health and healthcare across the globe that demand prompt action.

There is a well‑established bidirectional relationship between HIV and HBV. People living with HIV (PLHIV) face an elevated risk of acquiring HBV infection, and the reverse is equally true [4]. In sub‑Saharan Africa, between one and five out of every ten individuals with HIV have chronic HBV, and research shows that the immune suppression in PLHIV promotes HBV replication, reactivation, and a markedly faster progression up to five times higher to cirrhosis and liver cancer [5]. Moreover, HIV doubles the risk of mother-to-child transmission of HBV, further revealing the multidimensional effect of HIV on HBV dynamics [6]. Conversely, chronic HBV drives systemic inflammation and immune activation, thereby accelerating HIV disease progression. As a result, individuals with HIV/HBV co‑infection often experience rapid and worsening clinical outcomes, marked by multiple morbidities and increased mortality [7–9]. This dual burden complicates clinical management and imposes a significant toll on overall health and well‑being.

Like many LMICs, Tanzania faces the dual challenge of HIV and HBV coinfections. Current national data indicate that approximately 4.4% of Tanzanians are living with HIV, and more than 8% of these individuals are coinfected with HBV, placing the country in the intermediate‑burden category for these infections [10,11].

A clear understanding of both the magnitude of the problem and the determinants driving HIV/HBV coinfection is essential for developing targeted and cost‑effective interventions. Beyond the biological mechanisms already described, available evidence links this dual burden to specific behavioral and sociodemographic factors, notably male sex, rural residence, low socioeconomic status, multiple sexual partnerships, and co‑existing sexually transmitted infections (STIs) [12–15]. However, population‑level data on both prevalence and risk factors in Tanzania remain scarce. This evidence gap undermines accurate risk perception among policymakers and communities, and may hinder the design and implementation of strategic, evidence‑informed prevention and management programmes capable of mitigating the health and socioeconomic toll of HIV/HBV coinfection.

This study leverages the population based THIS 2022 dataset to examine and characterize the burden of HIV/HBV co-infections and its determinants in the general population. The evidence will generate a comprehensive epidemiologic profile that can inform national policy in Tanzania and countries with similar burdens while paving ways for integrated service delivery.

## Materials and methods

### Study design

This study involved secondary analysis of relevant data from the 5^th^ round of the Tanzania HIV Impact Survey (THIS). The survey was a nationwide household-based cross-sectional HIV survey that was conducted in Mainland Tanzania and Zanzibar between November 2022 and March 2023. A two-stage cluster randomized sampling technique was adopted. This entailed sequential selection of census enumeration areas (EAs) and the households within each EA. The sampling frame comprised all EAs in Tanzania as determined in the 2022 Population and Housing Census data. This included 104,188 EAs and 14,966,262 households. The enumeration areas (EAs) were selected using probability proportional to size (PPS) and stratified by region. Due to wide regional variation in HIV prevalence ranging from 0.2% to 11%, the sampling design was modified by categorizing regions into three tiers: low (<3%), intermediate (3–5.9%), and high (>6%) prevalence. On the other hand, approximately 35 households were randomly selected per EA using PPS.

### Study population

In the primary study, all individuals, both permanent residents and visitors, aged 15 years and above, who spent a night at the household before the survey were eligible for participation. Our secondary analysis included participants who were interviewed in the primary study and whose results for HIV and HBV testing were available. The dataset used for this analysis was accessed for research purposes on 31 July 2025. The data provided for analysis were de-identified, and the authors did not have access to information that could identify individual participants during or after data collection.

### Variables and measurements

THIS comprises three interlinked datasets: the household roster, the adult individual dataset, and the biomarker dataset. For the present analysis, only the adult individual and biomarker datasets were utilized. The household dataset was not included, as it did not contain variables directly relevant to the study objectives. Variables were selected a priori based on their availability across the relevant datasets and their established relevance in the literature on HIV, HBV, and HIV/HBV coinfection. From the adult individual dataset, we extracted demographic and behavioral characteristics, including age (continuous), sex (male/female), wealth quintile (lowest to highest), marital status (single, married, divorced, widowed), and level of education (no formal education through college). Behavioral and clinical exposure variables included age at sexual debut (<18/ ≥ 18), number of lifetime sexual partners (1, > 1), history of HBV vaccination (yes/no), chronic alcohol use (yes/no), and reported history of dialysis, blood transfusion, surgery or dental procedures, and body piercing or scarification (yes/no).

Diagnostic outcomes for HIV and HBV infection were obtained from the biomarker dataset and merged with the adult individual dataset to generate binary indicators (positive/negative) for each infection. These biomarker-confirmed outcomes formed the basis for defining HIV infection status, HBV infection status, and HIV/HBV coinfection. HIV testing followed a sequential rapid testing algorithm using SD Bioline™ HIV-1/2 as the screening test and Uni-Gold™ for confirmation; discrepant results were resolved using the Geenius™ HIV 1/2 Supplemental Assay and HIV-1 Total Nucleic Acid PCR. HBV testing was performed on plasma samples, with Abbott Architect Ci4100 used to screen for anti-HBc antibodies; anti-HBc-positive samples were further tested for HBsAg using SD Bioline HBsAg rapid test, with dual positivity indicating acute or chronic HBV infection and anti-HBc positivity alone indicating past or resolved infection.

### Data analysis

The analytical approach was guided by the Social Determinants of Health (SDH) framework, which conceptualizes health outcomes as the result of structured social, economic, and behavioral processes operating across multiple levels [16]. Within this framework, demographic and socioeconomic characteristics were treated as upstream structural determinants, behavioral and healthcare-related exposures as intermediate pathways, and HIV and HBV infection status as proximal biological outcomes.

Data were cleaned, manipulated, and analyzed using R version 4.4.2. All analyses accounted for the survey design and incorporated Jackknife replicate weights to minimize bias and ensure equitable representation of all participants. Participants with missing data on key variables were excluded from the respective analyses. In examining the relationship between the outcome and the independent variable, individuals with missing data on some values of the independent variable were also excluded.

A descriptive analysis was conducted whereby categorical variables were summarized using frequencies and proportions, and continuous variables using median and interquartile range. The primary outcome was a binary variable representing HIV/HBV coinfection status (Yes/No). This was derived from HIV and HBV test results: individuals testing positive for both infections were classified as coinfection-Yes (positive), while all others were classified as coinfection-No (Negative). To describe the distribution of the study variables by HBV/HIV coinfection status, a Rao-Scott chi-square test that adjusts for the study design was used.

Associations between the outcome and independent variables were determined using bivariable and multivariable logistic regression, and effect sizes were estimated in odds ratios. In bivariable analysis, all independent variables were included. For the multivariable analysis, variables with a bivariable p-value ≤ 0.20 were retained. Two models were built for multivariable analysis. The first model adjusted for sociodemographic factors, and the second model for behavioral factors. A statistical significance for the results was set at p ≤ 0.05 and 95% confidence intervals.

### Ethical considerations

This study involved secondary analysis of anonymized, population-based data collected during the 2022 Tanzania HIV Impact Survey (THIS), which received ethical approval from the National Institute for Medical Research (NIMR) and the Zanzibar Health Research Institute (ZAHRI). The original survey followed the ethical principles of the 1964 Declaration of Helsinki and its later amendments. All emancipated minors aged 15 years and adults aged 18 and above provided informed verbal consent. For the non-emancipated minors, assent was obtained from this group, while their parents or legal guardians provided consent for their participation. For this analysis, no direct contact with participants was involved, and all identifiers were removed prior to access. The reference number NIMR/HQ/R.8a/Vol.IX/4125.

## Results

The final analysis included 33,263 participants after removing those who did not consent to biomarker testing (2,294), and those with missing HBV infection results (400).

### Participants characteristics

A total of 33,263 participants were included in the analysis. The majority, 20,723 (61.6%), were adults, aged 25–64. Females comprised 19,037 (56.6%) of the study population, and most participants were in a marital union, 19,596 (58.3%). More than half of the participants, 19,529 (58.1%), had primary education, and 22,376 (66.6%) resided in rural areas and 18,283 (54.4%), reported having had more than one lifetime sexual partner. Only 200 (0.6%) of the participants reported having received the HBV vaccine (Table 1).

**Table 1:** Distribution of Sociodemographic characteristics of participants by HBV/HIV coinfection status n = 33,263.

	HBV/ HIV coinfection			
Characteristic	Overall	No n = 33,157 (%)	Yes n = 106 (%)	P value^1^
**Age (years)**				<0.001
15 - 24	9,818	9,815 (99.96)	3 (0.04)	
25 - 64	20,723	20, 624 (99.58)	99 (0.42)	
65 - 80	2,722	2,718 (99.89)	4 (0.11)	
**Sex**				0.294
Male	14,226	14,184 (99.77)	42 (0.23)	
Female	19,037	18,973 (99.69)	64 (0.31)	
**Marital status**				0.213
In union	19,596	19,540 (99.76)	56 (0.24)	
Not in union	13,607	13,557 (99.68)	50 (0.32)	
**Highest level of education**				0.009
No education	5,146	5,122 (99.51)	24 (0.49)	
Primary level	19,529	19,457 (99.71)	72 (0.29)	
Secondary and above level	8,559	8,549 (99.86)	10 (0.14)	
**Wealth quintile**				0.506
Lowest	7,432	7,407 (99.68)	25 (0.32)	
Second	7,661	7,633 (99.65)	28 (0.35)	
Middle	7,496	7,470 (99.77)	26 (0.23)	
Fourth	5,724	5,706 (99.73)	18 (0.27)	
Highest	4,937	4,928 (99.82)	9 (0.18)	
**Residence**				0.594
Urban	10,887	10,851(99.71)	36 (0.29)	
Rural	22,376	22,306 (99.74)	70 (0.26)	
**Age at which had sex first time (years)**				0.987
< 18	15,447	15,399 (99.72)	48 (0.28)	
≥ 18	15,561	15,504 (99.72)	57 (0.28)	
**Number of lifetime sexual partners**				<0.001
1	9,680	9,669 (99.92)	11 (0.08)	
> 1	18,283	18,194 (99.60)	89 (0.40)	
**Received Hepatitis B vaccine**				0.559
Yes	200	200 (1.00)	0 (0.00)	
No	32,688	32,583 (99.73)	105 (0.27)	
**History of blood transfusion or transplant**				0.494
Yes	1,742	1,735 (99.61)	7 (0.39)	
No	31,392	31,293 (99.73)	99 (0.27)	
**History of surgical procedures**				
Yes	6,210	6,177 (99.68)	24 (0.32)	0.485
No	27,033	26,951 (99.74)	82 (0.26)	
**History of dialysis**				
Yes	76	76 (100.00)	0 (0.00)	0.694
No	33,138	33,032 (99.73)	106 (0.27)	
**History of body piercing**				
Yes	15,217	15,164 (99.67)	53 (0.33)	0.145
No	18,031	17,978 (99.77)	53 (0.23)	
**Has tattoos**				
Yes	2,808	2,798 (99.80)	10 (0.20)	0.377
No	30,371	30,275 (99.72)	96 (0.28)	

^1^Rao Scott chi square test, 95% CI: 95% Confidence interval, PLWHIV: People Living With HIV, * n = 1832.

The HIV/HBV co-infection was significantly higher among persons aged 25–64, 99 (0.42%), compared to the age group of 15–24 years, 9,815 (0.04%), and 65–80 years, 4 (0.11%), p < 0.001. The prevalence of HIV/HBV co-infection was also significantly higher among participants with more than one sexual lifetime partner 89 (0.40%) compared to those with one sexual partner 11 (0.08%), p < 0.001 (Table 1)

### The prevalence of HIV/HBV coinfection

In the general population, the prevalence of HIV/HBV coinfection was 0.27% [95% CI: (0.21–0.34)], while among PLWHIV, the prevalence of HBV was 6.22% [(95% CI: (4.89–7.78)]. Additionally, among all individuals who participated in the study, 4.38% [95% CI (4.07–4.70)] were HIV positive, and 3.53% [95% CI: (3.26–3.82)] had HBV infection (Table 2).

**Table 2:** The prevalence of HIV, HBV, and HIV/HBV co-infections among participants (n = 33,263).

Characteristics	Unweighted Frequency	Weighted % (95% CI)
**HIV status**		
Positive	1,832	4.38 (4.07–4.70)
Negative	31,431	95.62 (95.30–95.93)
**HBV status**		
Positive	1,187	3.53 (3.26–3.82)
Negative	32,076	96.49 (96.18–96.74)
**HBV among PLWHIV** ^*^		
Positive	106	6.22 (4.89–7.78)
Negative	1,726	93.78 (96.18–96.74)
**HIV/HBV co-infection**		
Yes	106	0.27 (0.21–0.34)
No	33,157	99.73 (99.66–99.79)

PLWHIV: People living with HIV, * n < 33,263.

In multivariable logistic regression analysis, age remained independently associated with HIV/HBV coinfection. Relative to the 15–24 age group, the odds of HIV/HBV coinfection among participants aged 25–64 years was higher by 38% (aOR = 1.38(1.56–122.14; P = 0.019). Moreover, participants who were not in union were 2.63 times as likely to have HIV/HBV coinfection compared with those in union (aOR = 2.63; 95% CI: 1.68–4.15; p < 0.001). The protective association observed for educational attainment in the univariable analysis was attenuated and did not remain statistically significant after adjustment for other covariates. Having one lifetime sexual partner remained a strongly protective factor against the HIV/HBV coinfection (aOR = 0.19; 95% CI: 0.09–0.42; p < 0.001) (Table 3).

**Table 3:** Factors associated with HIV/HBV co-infection.

	Bivariate logistic regression		Multivariable logistic regression	
Characteristic	uOR (95% CI)	P – value	aOR (95% CI)	P – value
**Age (Years)**				
15 - 24	Ref			
25–64	10.37 (1.1–97.12)	0.040	1.38 (1.56–122.14)	0.019
65–80	2.66 (0.23–31.15)	0.434	2.07 (0.19–2.29)	0.552
**Sex**				
Male	Ref			
Female	1.32 (0.78–2.23)	0.305	1.12 (0.68–1.83)	0.655
**Marital status**				
In union	Ref			
Not in union	1.33 (0.85–2.09)	0.218	2.63 (1.68–4.15)	<0.001
**Education level**				
No education	Ref			
Primary level	0.58 (0.34–0.98)	0.041	0.63 (0.35–1.11)	0.110
Secondary level and above	0.29 (0.12–0.72)	0.008	0.43 (0.17–1.05)	0.063
**Wealth quintile**				
Lowest	Ref			
Second	1.09 (0.56–2.11)	0.805	1.08 (0.56–2.08)	0.820
Middle	0.72 (0.39–1.35)	0.306	0.68 (0.36–1.28)	0.232
Fourth	0.86 (0.41–1.80)	0.690	0.68 (0.25–1.83)	0.443
Highest	0.57 (0.23–1.40)	0.221	0.44 (0.16–1.24)	0.121
**Residence**				
Urban	Ref			
Rural	0.88 (0.53–1.44)	0.603	0.59 (0.30–1.18)	0.134
**Age at which had sex first time (years)**				
< 18	Ref			
≥ 18	1.00 (0.64–1.56)	0.988	0.75 (0.46–1.22)	0.243
**Number of sexual partners in lifetime**				
> 1	Ref			
1	0.19 (0.09–0.42)	<0.001	0.18 (0.08–0.41)	<0.001
**Surgical procedures**				
No	Ref			
Yes	1.21 (0.70–2.11)	0.493	1.29 (0.71–2.35)	0.394
**History of dialysis**				
No	Ref			
Yes	0.00 (0.00–91.85)	0.156	0.00 (0.00–90.06)	0.156
**History of blood transfusion**				
No	Ref			
Yes	1.48 (0.38–5.76)	0.57	0.57 (0.23–1.44)	0.236
**History of body piercing**				
No	Ref			
Yes	1.43 (0.88–2.32)	0.152	1.65 (1.01–2.68)	0.044
**Has tattoos**				
No	Ref			
Yes	0.70 (0.31–1.59)	0.393	0.65 (0.28–1.53)	0.320

uOR: Unadjusted Odds Ratio, aOR: Adjusted Odds Ratio, a: model I (Sociodemographic determinants), b: model 2 (risky behavior determinants).

### Sub-analysis of factors associated with HIV/HBV coinfection among PLWHIV

Among PLWHIV, only the number of sexual partners in one’s lifetime showed statistical significance in the bivariable analysis, with those with more than one partner being more likely to have HIV/HBV coinfection (P = 0.009). There was evidence that participants aged 25–64 years and males were more likely to have HIV/HBV coinfection; however, the associations were not statistically significant (Table 4).

**Table 4:** Distribution of Sociodemographic characteristics of participants by HBV/HIV coinfection among PLWHIV n = 1,832.

	HBV/ HIV coinfection			
Characteristic	Overall n = 1,832	No n = 1,726 (%)	Yes n = 106 (%)	P value^1^
**Age (years)**				0.403
15 - 24	115	112 (95.82)	3 (4.18)	
25 - 64	1,604	1,505 (93.36)	99 (6.64)	
65 - 80	113	109 (97.34)	4 (2.66)	
**Sex**				0.190
Male	549	507 (92.31)	42 (7.69)	
Female	1,283	1,219 (94.51)	64 (5.49)	
**Marital status**				0.684
In union	954	898 (94.05)	56 (5.95)	
Not in union	876	826 (93.48)	50 (6.52)	
**Highest level of education**				0.202
No education	308	284 (90.87)	24 (9.13)	
Primary level	1,281	1,209 (94.52)	72 (5.48)	
Secondary and above level	240	230 (93.58)	10 (6.42)	
**Wealth quintile**				0.601
Lowest	339	314 (92.14)	25 (7.86)	
Second	445	417 (92.73)	28 (7.27)	
Middle	448	422 (94.47)	26 (5.53)	
Fourth	373	355 (94.69)	18 (5.31)	
Highest	227	218 (95.20)	9 (4.80)	
**Residence**				0.617
Urban	672	636 (94.20)	36 (5.80)	
Rural	1,160	1,090 (93.45)	70 (6.55)	
**Age at which had sex first time (years)**				0.235
< 18	737	689 (92.52)	48 (7.48)	
≥ 18	963	906 (94.21)	57 (5.79)	
**Number of lifetime sexual partners**				0.009
1	345	334 (97.17)	11 (2.83)	
> 1	1,311	1,222 (92.87)	89 (7.13)	
**Received Hepatitis B vaccine**				0.481
Yes	9	9 (100.00)	0 (0.00)	
No	1,802	1,090 (93.74)	105 (6.26)	
**History of blood transfusion or transplant**				0.935
Yes	141	134 (94.01)	7 (5.99)	
No	1,684	1,585 (93.74)	99 (6.26)	
**History of surgical procedures**				
Yes	446	422 (94.73)	24 (5.27)	0.414
No	1,382	1,300 (93.48)	84 (6.52)	
**History of dialysis**				
Yes	6	6 (100.00)	0 (0.00)	0.649
No	1,824	1,718 (93.74)	106(6.26)	
**History of body piercing**				
Yes	1,002	949 (94.03)	53 (5.97)	0.715
No	830	777 (93.49)	53 (6.51)	
**Has tattoos**				
Yes	167	157 (96.00)	10 (4.00)	0.205
No	1,662	1,566 (93.54)	96 (6.46)	

^1^Rao Scott chi square test, 95% CI: 95% Confidence interval, PLWHIV: People Living With HIV, * n = 1832.

In the univariable analysis, the primary level of education, history of dialysis and number of sexual partners in lifetime showed statistical significance. After adjusting for other covariates, only the number of sexual partners in lifetime retained its statistical significance. Relative to having more than one sexual partner, having one or no sexual partner was associated with 58% reduced odds of HIV/HBV coinfection (aOR:0.42 (0.10–0.94), P = 0.035) (Table 5).

**Table 5:** Factors associated with HIV/HBV co-infection among PLWHIV.

	Bivariate logistic regression		Multivariable logistic regression	
Characteristic	uOR (95% CI)	P – value	aOR (95% CI)	P – value
**Age (Years)**				
15 - 24	Ref			
25–64	1.63 (0.17–15.46)	0.669	1.71 (0.21–14.17)	0.618
65–80	0.63 (0.05–7.6)	0.712	0.55 (0.05–5.92)	0.620
**Sex**				
Male	Ref			
Female	0.70 (0.40–1.21)	0.199	0.65 (0.37–1.12)	0.121
**Marital status**				
In union	Ref			
Not in union	1.10 (0.68–1.78)	0.686	1.30 (0.81–2.08)	0.278
**Education level**				
No education	Ref			
Primary level	0.58 (0.34–0.99)	0.047	0.58 (0.32–1.07)	0.083
Secondary level and above	0.68 (0.27–1.73)	0.420	0.79 (0.32–1.97)	0.610
**Wealth quintile**				
Lowest	Ref			
Second	0.92 (0.47–1.80)	0.805	0.99 (0.49–2.00)	0.973
Middle	0.69 (0.37–1.28)	0.232	0.76 (0.39–1.48)	0.416
Fourth	0.66 (0.32–1.37)	0.260	0.69 (0.26–1.88)	0.470
Highest	0.59 (0.23–1.49)	0.262	0.60 (0.22–1.65)	0.319
**Residence**				
Urban	Ref			
Rural	1.14 (0.68–1.92)	0.624	0.89 (0.44–1.78)	0.740
**Age at which had sex first time (years)**				
< 18	Ref			
≥ 18	0.76 (0.48–1.20)	0.239	0.66 (0.39–1.10)	0.111
**Number of sexual partners in lifetime**				
> 1	Ref			
≤ 1	0.38 (0.17–0.84)	0.017	0.42 (0.10–0.94)	0.035
**Surgical procedures**				
No	Ref			
Yes	0.80 (0.46–1.39)	0.421	0.96 (0.54–1.69)	0.881
**History of dialysis**				
No	Ref			
Yes	0.00 (0.00–0.16)	0.023	0.00 (0.00–1.25)	0.054
**History of blood transfusion**				
No	Ref			
Yes	0.95 (0.24–3.74)	0.945	0.45 (0.18–1.15)	0.100
**History of body piercing**				
No	Ref			
Yes	1.10 (0.66–1.83)	0.718	1.09 (0.66–1.81)	0.733
**Has tattoos**				
No	Ref			
Yes	0.60 (0.27–1.35)	0.219	0.64 (0.27–1.51)	0.7334

uOR: Unadjusted Odds Ratio, aOR: Adjusted Odds Ratio, a: model I (Sociodemographic determinants), b: model 2 (risky behavior determinants).

## Discussion

The burdens of HIV and HBV are still significant health challenges, affecting 4.38% and 3.53% of the population respectively in Tanzania. Strikingly, about seven out of every 100 individuals living with HIV had HBV infection, underscoring the disproportionately high burden in this subpopulation. Additionally, 0.27% of the general population had HIV-HBV coinfection. Although small, it still implies an important dual burden.

Compared to a decade ago, Tanzania has seen a notable decline in HIV incidence, but to lesser extent, hepatitis B virus (HBV) infection [17,18]. These gains are largely attributable to expanded access to effective preventive and treatment interventions including HIV pre-exposure prophylaxis, scale-up of antiretroviral therapy, prevention of mother-to-child transmission interventions, and efforts targeting key and vulnerable populations [18,19]. Although hepatitis B vaccination is included in Tanzania’s routine childhood immunization schedule through the pentavalent vaccine, the hepatitis b birth dose is not part of the free routine immunization program, limiting prevention of vertical transmission. In adults, vaccination is not universally provided for the general population and is more commonly available among specific high-risk groups such as healthcare workers [20], which may contribute to persistent HBV transmission and chronic infection [21]. In this context therefore, there is a slow decline of this specific burden, and it continues to be ranked relatively higher. The systematic reviews and regional studies estimating prevalence at about 7% [18]. This persistent burden highlights ongoing risks beyond HIV transmission including age and multiple sexual partnerships, which were significantly associated with HIV/HBV coinfection in this study. While unprotected sex remains a key driver, unsafe medical or cosmetic practices, and sharing of contaminated sharp instruments though not significant predictors in our study have been reported as important contributors to HBV transmission in other settings, underscoring the need for strengthened prevention and control strategies [22,23].

Unlike clinic-based studies that assess coinfection exclusively among known HIV-positive individuals, our population-level approach provides complementary insights into the overall burden of coinfection and supports the design of community-wide prevention strategies. Strengthening guidelines and integrating routine HBV screening, vaccination, and treatment into decentralized HIV services would improve outcomes, enhance cost-effectiveness, and align with international standards for comprehensive care [24].

The prevalence of HIV/HBV coinfection in this study (0.27%) is consistent with and slightly higher than previous findings among pregnant women in Tanzania where coinfection prevalence was reported (0.1%) [25], likely reflecting differences in population characteristics and exposure risk. Among HIV-positive participants, the coinfection prevalence of approximately 7% aligns closely with both the global estimate of 7.4% reported among PLHIV [26] and previously reported rates in Tanzania [7,8]. PLHIV face heightened risk of HBV-related disease progression, including accelerated liver fibrosis and increased hepatocellular carcinoma risk [27], making even this comparatively modest burden clinically significant. Even at low prevalence, however, the presence of this dual infection has important clinical and policy implications. It highlights the need for thorough investigation and tailored treatment strategies for patients with either infection, ensuring holistic management and reducing the risk of poor outcomes [28]. Moreover, the findings reinforce the urgency of policies and guidelines that prioritize routine screening, public awareness, and integrated preventive measures addressing both HIV and HBV [29,30]

In multivariable regression analysis, age remained a robust predictor after adjustment, suggesting that HIV/HBV coinfection reflects lifetime cumulative exposure. Older cohorts were less likely to have benefited from Tanzania’s childhood HBV vaccination program introduced in 2002 [31], leaving them unprotected during years of prolonged exposure to transmissions risks. Similar age gradients in HIV/HBV coinfection have been reported across sub-Saharan Africa, supporting the role of cohort effects and cumulative exposure in shaping coinfection patterns [32].

Marital status also showed an independent association, with individuals not in union having higher odds of coinfection. This likely reflects underlying sexual network dynamics, including partnership instability and increased likelihood of multiple or sequential partnerships. Multiple sexual partnerships likely increase exposure opportunities. In line with this, a recent Tanzanian antenatal survey found that history of multiple partners markedly raised the odds of HIV/HBV coinfection [33].

A stratified sub-analysis among the 1,832 PLHIV in this survey identified number of lifetime sexual partners as the sole independent predictor of HBV coinfection, with those reporting a single lifetime partner having significantly lower odds than those with multiple partners. No significant associations were observed for age, sex, education, wealth, residence, or medical and cosmetic exposure history. This finding reflects the well-established overlap in sexual transmission routes shared by HIV and HBV [34,35].

Importantly, in high-endemicity settings like Tanzania, chronic HBsAg positivity predominantly reflects early-life acquisition with an estimated risk of progression to chronic HBV infection are 90% for infections acquired perinatally, 30–60% for early childhood infections, and under 10% for HBV infection acquitted in adulthood [36]. The absence of significant sociodemographic predictors supports universal rather than risk-stratified HBV screening within HIV care and treatment centers, with tenofovir-containing regimens for coinfected individuals and hepatitis B vaccination for those non-immune, leveraging existing ART infrastructure to advance integrated viral hepatitis elimination goals [37,38].

These findings underscore the importance of integrated HIV and HBV prevention strategies that prioritize routine HBV screening among people living with HIV, particularly older adults and individuals with documented sexual risk histories. Beyond screening, prevention and treatment programmes should be designed to account for key demographic, socioeconomic, and behavioral determinants -including age, sex, place of residence, and socioeconomic status – to ensure that HIV–HBV interventions are both targeted and responsive to population-level risk patterns [39].

### Strength and limitation

The use of nationally representative survey data with biomarker confirmation of HIV and HBV status enhances the generalizability of our findings. The integration of behavioral, demographic, and clinical variables allows for robust multivariable modelling. However, limitations pertinent to this secondary analysis include the cross-sectional design, which precludes causal inference, and potential misclassification of exposures such as lifetime sexual partners, which are subject to recall bias.

## Conclusion

Despite substantial decline in burden of HIV and HBV, these comorbidities remain critical health challenges in Tanzania, affecting between 4–6 out of every 100 Tanzanians. Although overall coinfection prevalence is low, HIV/HBV coinfection remains an important concern in Tanzania, particularly among the age group of 15–64 years and those with multiple sexual partnerships. A disproportionately high prevalence of HBV among PLHIV highlights critical gaps in existing preventive, screening, and treatment programs. World Health Organization and national guidelines recommend periodic HBV testing for PLHIV to optimize antiretroviral therapy (ART) given that tenofovir-based regimes effectively suppress both HIV and HBV [40]. Future research should explore subgroup patterns of coinfection and assess the impact of integrated care models on clinical outcomes.
