# Supplementary material for: HIV and Hepatitis B co-infection in Tanzania: Analysis of the 2022–2023 Tanzania HIV Impact Survey
Source: PLoS One. 2026 Sep 11;21(9):e0358242. doi: 10.1371/journal.pone.0358242 (PMC13567782; doi:10.1371/journal.pone.0358242)
Supplement: Supplementary file 1 [file pone.0358242.s001.docx]

Felistar Mwakasungura,

P.O. Box 65001,

MUHAS.

26^th^ April 2026.

To,

Academic Editor,

PLOS One.

**RE: RESPONSES TO REVIEWERS COMMENTS**

**Dear Sir/Madam,**

Refer to the heading above, kindly find the below responses from the comments we received on our manuscript submit to your esteemed journal.

**Response to reviewer 1**

1. For the results, suggest first starting by describing the participant characteristics before presenting the HIV/HBV infection prevalence. Usually, results start with a general description of the study population before presenting the main outcomes.

**Response**: Thank you for this feedback. We accept this recommendation. We have reported participants' characteristics before presenting HIV/HBV coinfection prevalence Line 186 to 209.

2. Lines 182-184: the sentence is unclear and there is no mention of the age groups with highest HIV/HBV co-infection. It is unclear which age groups refer to the adult population, and which age groups refer to the younger population. In addition, adults are usually defined as people aged 18 and older which does not seem to be the definition that the authors are using. Recommend clearly including the age groups (25-64 years, 15-24 years, 65-80 years) that are being referred to in all sentences of this manuscript instead of mentioning adults, young and elderly. The use of the word “elderly” is not recommended, a better term is older adults.

**Response**: Thank you for this observation. We have incorporated clearly the age groups as per your recommendations Line 193 to 195.

3. Tables 2 and 3: For the analysis of healthcare risk factors, it is recommended to create separate variables because risks are different: receipt of blood transfusion; history of dialysis; receipt of transplant; exposure to surgical procedures. By lumping all those exposures together, authors might miss an important risk factor such as dialysis and blood transfusions.

**Response:** Thank you for your recommendation. We have separated the variables as recommended; however, due to a significantly high imbalance of the outcomes, we are getting very wide confidence intervals in some variables, such as history of dialysis. This was the reason we combined them in the first analysis. We have added the tables with separated variables as table 1& 3.

4. Table 3: recommend keeping age as categorical variable (15-24; 25-64; 65-80) to be consistent with the presentation in Table 2. In addition, revise the text that reports on age in multivariate analysis accordingly. I suspect that the main age group impacted by coinfection would be 15-64 years as shown in table 2. This is important especially to highlight in the discussion as they are the economically productive age groups and early deaths due to coinfection would impact economic productivity in Tanzania. Of note the lower prevalence in persons aged 65-80 is expected as most of the persons coinfected would have died earlier due to complications of their infections especially liver cirrhosis and cancer which tends to present earlier among people in Africa. https://pmc.ncbi.nlm.nih.gov/articles/PMC10204139/. <https://www.sciencedirect.com/science/article/pii/S2949916X24000112>

**Response:**  Thank you for your comment. We categorized the age into (15-24; 25-64; 65-80) age groups; however, due to an imbalance in the outcome of interest, we were getting very wide confidence intervals. We have added the tables with age in groups in Table 1 for your review.

5. In the discussion, the authors mention that the overall prevalence of HIV/HBV co-infection is low, but they also point out that HBV co-infection among persons living with HIV is almost 7% which is high. Given this finding, it would be more useful if the authors analyzed factors associated with HBV co-infection among persons with HIV compared to persons who do not have HIV rather than looking at factors associated with overall prevalence of coinfection in the population. Therefore, I would recommend that the authors incorporate in their bivariate and multivariate analysis, factors associated with being co-infected with HBV among people living with HIV. For programmatic planning and to inform policy makers, it would be more useful to approach it from that perspective rather than looking at overall prevalence of HIV/HBV co-infection in the population. Policy makers need more clarity on how to integrate HBV testing and treatment among persons with HIV and the analysis using HBV infection among persons with HIV would help present clear recommendations to policy makers in Tanzania.

**Response**: We sincerely thank the reviewer for this thoughtful and important comment. We agree that examining factors associated with HBV co-infection specifically among people living with HIV would provide valuable programmatic insights for HIV care integration and policy planning.

However, the primary objective of our study was to characterize the burden and determinants of HIV/HBV co-infection in the general population using nationally representative data, particularly because population-level evidence on HIV/HBV co-occurrence in Tanzania remains limited. While several studies have explored HBV prevalence among people living with HIV (<https://onlinelibrary.wiley.com/doi/full/10.1111/jvh.13217>, <https://www.frontiersin.org/journals/public-health/articles/10.3389/fpubh.2025.1565621/full>, <https://pmc.ncbi.nlm.nih.gov/articles/PMC5989175/>), there is comparatively less evidence describing the epidemiology of HIV/HBV coinfection at the population level, which was the main knowledge gap this study sought to address.

6. Discussion lines 215-216: while hepatitis B vaccine was introduced in children as part of the pentavalent vaccine in Tanzania in 2002, the hepatitis B birth dose is not available for free and is not part of the routine immunization program in Tanzania. As for adult hepatitis B vaccination, while it might be available and mainly targets healthcare workers, there is no recommendation for universal adult vaccination against hepatitis B in Tanzania and the vaccine is not free. Reference 21 that the authors cite is focused on healthcare workers and not the general population or persons with HIV. Authors need to revise this sentence to reflect the current availability of hepatitis B vaccine in Tanzania in newborns, children and adults. The lack of hepatitis B birth dose vaccine to prevent vertical transmission of HBV is not mentioned anywhere by the authors. Vertical transmission is a key risk factor for progression to chronic HBV infection. While some persons coinfected with HIV and HBV could have acquired HBV infection at birth, this cross-sectional survey cannot identify this risk.

**Response**: Thank you for this important clarification regarding hepatitis B vaccination policy and availability in Tanzania. I agree that the original statement was too broad and that Reference 21 primarily reflects vaccination coverage among healthcare workers rather than the general population. I have revised the discussion to better reflect the current hepatitis B vaccination context in Tanzania line 256 – 262.

7. Lines 219-221: data from this manuscript do not support those assumptions. The only risk factor in this study were age and number of sex partners. Medical procedures and cosmetic practices were not significant. In addition, for hepatitis B, the main risk factor for chronic HBV infection is vertical transmission. The risk of progression to chronic HBV infection after exposure is up to 90% in newborns and drops to 5% in persons older than 5 years. The authors never address this risk factor in their discussion which would miss a key intervention that would further prevent HBV infections including coinfections in the future.

**Response**: Thank you for this important observation. I agree that the original statement required clearer alignment with the findings of this study. In our analysis, the significant factors associated with HIV/HBV coinfection were age and number of sexual partners. Medical procedures and cosmetic practices were not statistically significant predictors in our dataset. The intention of the discussion was to place our findings within the broader context of existing literature, where other studies have identified additional contributors to HBV transmission, including unsafe medical procedures, cosmetic practices involving sharps, and other blood exposure risks. However, I agree that the wording in the original manuscript could be interpreted as if these factors were directly demonstrated by our data, we have revised it line 265 to 277.

8. Lines 225-227: the prevalence of hepatitis B along persons with HIV was almost 7% in this survey, so it does match what has been reported previously in Tanzania and is not lower than previous estimates. I think authors are confusing overall prevalence of coinfection in the general population with HBV coinfection among persons with HIV. Usually, all studies assess HBV coinfection among persons with HIV as it would be most relevant for policy decisions. Hence, my previous recommendation to the analysis to assess factors associated with HBV coinfection among persons with HIV rather than the general population.

**Response**: Thank you for this observation. Indeed, we acknowledge the distinction between HBV coinfection prevalence in the general population versus among persons living with HIV. We have clarified this distinction in the manuscript to avoid confusion and to better contextualize our findings within the broader literature line 279-284.

9. Revise lines 222-227 to incorporate some of the information with the next paragraph starting at line 228 as they are related. All literature looked at HBV infection among persons with HIV.

**Response**: Thank you for this suggestion. We have revised and merged the previously separate paragraphs at lines 222–227 and 228 into a single cohesive paragraph as recommended line 278 to 284

10. Line 233: need to add the importance of introducing hepatitis B birth dose to prevent vertical transmission of HBV.

**Response**: Thank you for this important recommendation. We have added on hepatitis B birth dose as a critical intervention on HBV line 288 to 293.

11. Lines 237-239: Ref 28 refers to people who inject drugs and should not be cited to compare to general population without context. The 7.4% (ref 27) refers to prevalence of hepatitis B among persons with HIV globally and is not much different than what the authors found in Tanzania. The discussion needs revision and several sections can be combined to summarize the prevalence of HBV infection among persons with HIV and link to suggested recommendations like screening all persons with HIV for hepatitis B and incorporating hepatitis B testing in HIV clinics as well as during antenatal care, providing hepatitis B vaccination to persons with HIV wo have not been exposed (total anti-HBc negative) and to newborns to prevent vertical transmission.

**Response**: Thank you for this observation. We have incorporated the correct references and revised the section, line 296 to 324.

12. Line 275: Older adults (aged 65 and older) do not have higher prevalence than adults aged 15-64 years. The authors need to use age groups to describe their data rather than saying older adults.

**Response:** Thank you for this observation. We have revised on the conclusion section on the age group as per recommendation line 372.

13. Ref 29 is missing authors. If it is referring to the WHO guidelines, the reference needs to be updated to the more recent guidelines published in 2024. <https://www.who.int/publications/i/item/9789240090903>

**Response:** Thank you for this observation. We have updated the references accordingly line 324.

14. Discussion and associated references could also be strengthened by including the guidance of incorporating HBV screening and treatment in HIV services. <https://www.who.int/publications/i/item/9789240119529>

**Response:** Thank you for this guidance. We have added the consolidated guidance in our discussion section line 330.

**Response to Reviewer 2**

1. Describe the laboratory testing methods: for screening and confirmation of HIV and HBV infection

**Response:** Thank you for this recommendation. We have added this part on our method section line 134 to 143.

2. Provide any details on prevalence among pregnant women if that data was collected...if not provide this in the limitations because elimination of mother to child HBV infection is top priority.

**Response:** Thank you for this observation. This study was designed to assess HBV/HIV coinfection in the general adult population, pregnancy-specific estimates were outside the scope of this study.

3. Discuss infant immunization program in Tanzania to help explain the low number of vaccinated individuals in the study 200 vs 32,688. They could account for the proportion of participants who should have benefited from the global immunization program initiative from the time it was rolled out in Tanzania. How is the coverage so far?

**Response:** Thank you for this observation. We have expanded the discussion to contextualize the low adult vaccination coverage within Tanzania's immunization history, noting that HBV vaccination was introduced through the pentavalent vaccine in 2002 targeting infants only, adult vaccination remains largely restricted to healthcare workers, and less to the general population has been screened for HBV line 257 to 262 and line 333.

4. In the discussion, include comparison with close neighbors such as Kenya and Uganda as part of regional data. for example, Kenya: <https://pubmed.ncbi.nlm.nih.gov/39541401/>

**Response:** Thank you for this recommendation. We have added statistics accordingly line 287.

Sincerely,

Felistar Mwakasungura, MD, MSc.
