# Supplementary material for: HIV and Hepatitis B co-infection in Tanzania: Analysis of the 2022–2023 Tanzania HIV Impact Survey
Source: PLoS One. 2026 Sep 11;21(9):e0358242. doi: 10.1371/journal.pone.0358242 (PMC13567782; doi:10.1371/journal.pone.0358242)
Supplement: Supplementary file 2 [file pone.0358242.s002.docx]

Felistar Mwakasungura,

P.O. Box 65001,

MUHAS.

7^th^ June 2026.

To,

Academic Editor,

PLOS One.

**RE: RESPONSES TO REVIEWERS COMMENTS**

**Dear Sir/Madam,**

Refer to the heading above, kindly find the below responses from the comments we received on our manuscript submit to your esteemed journal.

**Response to reviewer 1**

1. Table 2: weighted percentages for the negative and no category should also be presented rather than only showing % among positive and yes.

**Response**: Thank you for this feedback. We accept this recommendation. We have added the percentages accordingly Table 2, Line 229-230.

2. Table 3: while the confidence intervals might be large if age is presented as a categorical variable or exposure to certain medical procedures, this should not restrict the presentation of those data as it is important to analyze data by age group and to differentiate medical exposures. Based on Table 1, the sample size in each age category is more than enough to present data by age groups in Table 3. It is also inadequate to lump blood transfusion with transplant and dialysis and surgical procedures. Those categories should be separated into blood transfusion (findings inform safety of blood in the country), dialysis (high risk medical intervention for acquiring HBV and HCV), and then other medical procedures (including transplant).

**Response**: Thank you for your insightful feedback, we have incorporated the suggestion to refine the work accordingly, Line 214 and 241.

3. While authors are interested in overall population level prevalence of HIV/HBV co-infection, the results would not be useful from a programmatic and public health perspective as their data cannot be used to come up with key recommendations to improve HBV screening and treatment based on HIV status. Is coinfection driven by acquiring HIV in adulthood or acquiring HBV in adulthood? It seems that the coinfection is driven by the HIV infection in adults. HBV infection probably occurred in infancy and childhood. However, authors did not look at that to present key drivers of coinfection. Analyzing data looking at HBV coinfection among persons with HIV compared to those who do not have HIV has not been done at the population level in Tanzania and none of the papers cited used large scale nationally representative surveys like THIS. I would highly recommend the authors to reconsider that comment as it would not preclude them from presenting overall prevalence in the population as well. Hence, adding this analysis would help guide national viral hepatitis and HIV policies in Tanzania.

**Response:** Thank you for your recommendation. We have included the analysis of HIV/HBV among PLHIV in our study as a sub-analysis of the study. We have added a section in the discussion on the sub-analysis as well from line 346 – 357.

4. I would recommend revising the discussion to compile common information together and avoid redundancy. For example, lines 241-250 discuss HIV/HBV coinfection prevalence and similarly lines 259-269 discuss that same point. The ideas in those paragraphs can be combined and summarized in one paragraph. Lines 251-258 mix different ideas (hepB birth dose with integration of HBV in HIV services) and the data presented in this manuscript do not show how incorporating HBV screening and treatment in HIV clinics is useful without showing the difference in prevalence at the population level of HBV infection among persons with HIV compared to HBV infection among persons who do not have HIV.

**Response:**  Thank you for your comment. I have removed the section that creates redundancy. On Hep B vaccine this section has been removed to discuss on the findings on HIV/HBV in PLHIV.

5. The authors did not highlight that the main risk of HBV infection in high prevalence settings is perinatal and early childhood transmission. This is important to bring up as most people most likely acquired HBV infection in childhood due to MTCT and later acquired HIV infection through sexual transmission. The risk of progression to chronic HBV infection is highest among newborns and children aged <5 years. Only 5% of adults exposed to HBV progress to chronic HBV infection while up to 90% of newborns exposed to HBV progress to chronic HBV infection.

**Response**: We sincerely thank the reviewer for this thoughtful and important comment. We have explained this in the discussion Line 351-357

6. Reference list could be improved by removing ref 32 and 34 which are redundant and are an old guidance. This is the updated WHO guidance: <https://www.who.int/publications/i/item/9789240090903> Ref 35 needs an author.

**Response**: Thank you for highlighting this duplicate referencing, I have removed the duplicate and updated the current guideline of 2024 accordingly, Line 306 - 308.

7. Recommend authors proofread their manuscript for grammatical errors before submitting. Example lines 50-52 are incoherent. Data is usually a plural term as it contains more than one piece of information (line 142: data were cleaned rather than data was cleaned). Lines 183-185: do not need to say age groups of the population (revise to among persons aged 25-64 or among persons 25-64 years of age etc.). lines 265-269.

**Response**: Thank you for your feedback I have rectified the grammar on the areas you have highlighted and proof read the whole document.

Sincerely,

Felistar Mwakasungura, MD, MSc.
