# Supplementary material for: HIV and Hepatitis B co-infection in Tanzania: Analysis of the 2022–2023 Tanzania HIV Impact Survey
Source: PLoS One. 2026 Sep 11;21(9):e0358242. doi: 10.1371/journal.pone.0358242 (PMC13567782; doi:10.1371/journal.pone.0358242)
Supplement: Supplementary file 3 [file pone.0358242.s003.docx]

Felistar Mwakasungura,

P.O. Box 65001,

MUHAS.

3^rd^ August 2026.

To,

Academic Editor,

PLOS One.

**RE: RESPONSES TO REVIEWERS COMMENTS**

**Dear Sir/Madam,**

Refer to the heading above, kindly find the below responses from the comments we received on our manuscript submit to your esteemed journal.

**Response to reviewer 1**

1. Table 2: the negative category needs to also include weighted 95% confidence intervals. Or a better presentation would be to just present the positive/yes along with total denominator for each variable:

Example:

Characteristics Number/Total tested Weighted % (95% CI)

HIV positive 1832/ 33263 4.38 (4.07-4.70)

Hepatitis B positive 1187/33263 3.53 (3.26-3.82)

Hepatitis B among PLWHIV 106/1832 6.22 (4.89-7.78)

HIV/HBV coinfection 106/33263 0.27 (0.21-0.34)

**Response**: Thank you for this feedback. We accept this recommendation and updated table 2 accordingly.

2. Line 202-209: sentences need to be revised to match the results in Table 3. There is no 1.02 in Table 3. Looks like persons aged 25-64 years had higher odds of HIV/HBV coinfection than those aged 15-24 years. Same for other numbers presented in the text which do not match numbers in Table 3.

**Response**: Thank you for your insightful feedback, we have incorporated the suggestion to refine the work accordingly, Line 238 and 246.

3. Discussion needs to be improved by focusing on one idea per paragraph. For example, lines 254-261 and 287-298 cover multiple topics which confuses the reader.

**Response:** Thank you for your recommendation. We have separated the paragraphs for the reader to get more clarity.

4. Manuscript needs to be proofread for clarity and English and grammar. All acronyms are defined on first use and lines 183-186 are still unclear.

**Response:**  Thank you for your comment. We have updated the section Line 188 to Line 192. We have also proofread to check for grammar.

Sincerely,

Felistar Mwakasungura, MD, MSc.
