# Supplementary material for: HIV and Hepatitis B co-infection in Tanzania: Analysis of the 2022–2023 Tanzania HIV Impact Survey
Source: PLoS One. 2026 Sep 11;21(9):e0358242. doi: 10.1371/journal.pone.0358242 (PMC13567782; doi:10.1371/journal.pone.0358242)
Supplement: Peer review history

# Peer review: Decision Letter 0

**DOI:** 10.1371/journal.pone.0358242.r001

**Authors:** Hans L. Tillmann

16 Apr 2026

Dear Dr. Mwakasungura,

plosone@plos.org. When you're ready to submit your revision, log on to https://www.editorialmanager.com/pone/ and select the 'Submissions Needing Revision' folder to locate your manuscript file.

- A letter that responds to each point raised by the academic editor and reviewer(s). You should upload this letter as a separate file labeled 'Response to Reviewers'.
- A marked-up copy of your manuscript that highlights changes made to the original version. You should upload this as a separate file labeled 'Revised Manuscript with Track Changes'.
- An unmarked version of your revised paper without tracked changes. You should upload this as a separate file labeled 'Manuscript'.

If you would like to make changes to your financial disclosure, please include your updated statement in your cover letter. Guidelines for resubmitting your figure files are available below the reviewer comments at the end of this letter.

If applicable, we recommend that you deposit your laboratory protocols in protocols.io to enhance the reproducibility of your results. Protocols.io assigns your protocol its own identifier (DOI) so that it can be cited independently in the future. For instructions see: https://journals.plos.org/plosone/s/submission-guidelines#loc-laboratory-protocols. Additionally, PLOS ONE offers an option for publishing peer-reviewed Lab Protocol articles, which describe protocols hosted on protocols.io. Read more information on sharing protocols at https://plos.org/protocols?utm_medium=editorial-email&utm_source=authorletters&utm_campaign=protocols.

As the corresponding author, your ORCID iD is verified in the submission system and will appear in the published article. PLOS supports the use of ORCID, and we encourage all coauthors to register for an ORCID iD and use it as well. Please encourage your coauthors to verify their ORCID iD within the submission system before final acceptance, as unverified ORCID iDs will not appear in the published article. *Only*  the individual author can complete the verification step; PLOS staff *cannot*  verify ORCID iDs on behalf of authors.

We look forward to receiving your revised manuscript.

Kind regards,

Hans L. Tillmann

Academic Editor

PLOS One

Journal Requirements:

When submitting your revision, we need you to address these additional requirements.

1.Please ensure that your manuscript meets PLOS ONE's style requirements, including those for file naming. The PLOS ONE style templates can be found at

https://journals.plos.org/plosone/s/file?id=wjVg/PLOSOne_formatting_sample_main_body.pdf and

https://journals.plos.org/plosone/s/file?id=ba62/PLOSOne_formatting_sample_title_authors_affiliations.pdf

2. Please include a caption for figure 1.

3. If the reviewer comments include a recommendation to cite specific previously published works, please review and evaluate these publications to determine whether they are relevant and should be cited. There is no requirement to cite these works unless the editor has indicated otherwise.

Additional Editor Comments:

The suggested points to address will improve you manuscript and make it more compatative, and I believe, you can address all reviewer's comments.

[Note: HTML markup is below. Please do not edit.]

Reviewers' comments:

Reviewer's Responses to Questions

**Comments to the Author**

1. Is the manuscript technically sound, and do the data support the conclusions?

Reviewer #1: Yes

Reviewer #2: Yes

**********

2. Has the statistical analysis been performed appropriately and rigorously?

Reviewer #1: No

Reviewer #2: Yes

**********

3. Have the authors made all data underlying the findings in their manuscript fully available?

The PLOS Data policy

Reviewer #1: Yes

Reviewer #2: No

**********

4. Is the manuscript presented in an intelligible fashion and written in standard English?

Reviewer #1: No

Reviewer #2: Yes

**********

Reviewer #1: General comments:

• In this paper, authors analyzed factors associated with HIV and HBV coinfection in Tanzania using available data from a nationally representative survey conducted in 2022-2023. The survey methods were robust and ensure that findings are representative of the general population in Tanzania.

• The manuscript overall addresses an important topic. In order to strengthen the analysis and generate findings that can be used by policy makers, I would suggest that the authors incorporate in their analysis, factors associated with HBV infection among persons with HIV rather than only looking at factors associated with overall prevalence of HIV/HBV coinfection in the population. This would follow the usual trend in analyzing such data and can be compared to existing global and regional studies. Policy makers need more clarity on how to integrate HBV testing and treatment among persons with HIV. Therefore, analyzing the data to look at factors associated with HBV infection among persons with HIV would help present clear recommendations to policy makers in Tanzania.

• The discussion specifically needs to be significantly revised to avoid repetitions, compare similar populations and present comprehensive suggestions to diagnose, manage and prevent HIV/HBV coinfections in Tanzania. It is also important that the authors incorporate the role of hepatitis B vaccination in newborns and children to prevent HBV infection. The risk of progression to chronic HBV infection is highest among newborns and children aged <5 years. Only 5% of adults exposed to HBV progress to chronic HBV infection while up to 90% of newborns exposed to HBV progress to chronic HBV infection. Tanzania only provides hepatitis B vaccine for free for children at the age of 6, 10 and 14 weeks (https://immunizationdata.who.int/dashboard/regions/african-region/TZA). Hepatitis B birth dose which prevents vertical transmission has not been introduced. Vertical transmission is the main reason for HBV infections in Tanzania and most of Sub-Saharan Africa. While this survey could not assess MTCT as a risk factor due to the cross-sectional nature, it is expected that many of the persons with HBV were infected at birth or in early childhood (https://www.thelancet.com/journals/langas/article/PIIS2468-1253(25)00375-9/fulltext).

Specific comments:

• For the results, suggest first starting by describing the participant characteristics before presenting the HIV/HBV infection prevalence. Usually, results start with a general description of the study population before presenting the main outcomes.

• Lines 182-184: the sentence is unclear and there is no mention of the age groups with highest HIV/HBV co-infection. It is unclear which age groups refer to the adult population, and which age groups refer to the younger population. In addition, adults are usually defined as people aged 18 and older which does not seem to be the definition that the authors are using. Recommend clearly including the age groups (25-64 years, 15-24 years, 65-80 years) that are being referred to in all sentences of this manuscript instead of mentioning adults, young and elderly. The use of the word “elderly” is not recommended, a better term is older adults.

• Tables 2 and 3: For the analysis of healthcare risk factors, it is recommended to create separate variables because risks are different: receipt of blood transfusion; history of dialysis; receipt of transplant; exposure to surgical procedures. By lumping all those exposures together, authors might miss an important risk factor such as dialysis and blood transfusions.

• Table 3: recommend keeping age as categorical variable (15-24; 25-64; 65-80) to be consistent with the presentation in Table 2. In addition, revise the text that reports on age in multivariate analysis accordingly. I suspect that the main age group impacted by coinfection would be 15-64 years as shown in table 2. This is important especially to highlight in the discussion as they are the economically productive age groups and early deaths due to coinfection would impact economic productivity in Tanzania. Of note the lower prevalence in persons aged 65-80 is expected as most of the persons coinfected would have died earlier due to complications of their infections especially liver cirrhosis and cancer which tends to present earlier among people in Africa. https://pmc.ncbi.nlm.nih.gov/articles/PMC10204139/. https://www.sciencedirect.com/science/article/pii/S2949916X24000112

• In the discussion, the authors mention that the overall prevalence of HIV/HBV co-infection is low, but they also point out that HBV co-infection among persons living with HIV is almost 7% which is high. Given this finding, it would be more useful if the authors analyzed factors associated with HBV co-infection among persons with HIV compared to persons who do not have HIV rather than looking at factors associated with overall prevalence of coinfection in the population. Therefore, I would recommend that the authors incorporate in their bivariate and multivariate analysis, factors associated with being co-infected with HBV among people living with HIV. For programmatic planning and to inform policy makers, it would be more useful to approach it from that perspective rather than looking at overall prevalence of HIV/HBV co-infection in the population. Policy makers need more clarity on how to integrate HBV testing and treatment among persons with HIV and the analysis using HBV infection among persons with HIV would help present clear recommendations to policy makers in Tanzania.

• Discussion lines 215-216: while hepatitis B vaccine was introduced in children as part of the pentavalent vaccine in Tanzania in 2002, the hepatitis B birth dose is not available for free and is not part of the routine immunization program in Tanzania. As for adult hepatitis B vaccination, while it might be available and mainly targets healthcare workers, there is no recommendation for universal adult vaccination against hepatitis B in Tanzania and the vaccine is not free. Reference 21 that the authors cite is focused on healthcare workers and not the general population or persons with HIV. Authors need to revise this sentence to reflect the current availability of hepatitis B vaccine in Tanzania in newborns, children and adults. The lack of hepatitis B birth dose vaccine to prevent vertical transmission of HBV is not mentioned anywhere by the authors. Vertical transmission is a key risk factor for progression to chronic HBV infection. While some persons coinfected with HIV and HBV could have acquired HBV infection at birth, this cross-sectional survey cannot identify this risk.

• Lines 219-221: data from this manuscript do not support those assumptions. The only risk factor in this study were age and number of sex partners. Medical procedures and cosmetic practices were not significant. In addition, for hepatitis B, the main risk factor for chronic HBV infection is vertical transmission. The risk of progression to chronic HBV infection after exposure is up to 90% in newborns and drops to 5% in persons older than 5 years. The authors never address this risk factor in their discussion which would miss a key intervention that would further prevent HBV infections including coinfections in the future.

• Lines 225-227: the prevalence of hepatitis B along persons with HIV was almost 7% in this survey, so it does match what has been reported previously in Tanzania and is not lower than previous estimates. I think authors are confusing overall prevalence of coinfection in the general population with HBV coinfection among persons with HIV. Usually, all studies assess HBV coinfection among persons with HIV as it would be most relevant for policy decisions. Hence, my previous recommendation to the analysis to assess factors associated with HBV coinfection among persons with HIV rather than the general population.

• Revise lines 222-227 to incorporate some of the information with the next paragraph starting at line 228as they are related. All literature looked at HBV infection among persons with HIV.

• Line 233: need to add the importance of introducing hepatitis B birth dose to prevent vertical transmission of HBV.

• Lines 237-239: Ref 28 refers to people who inject drugs and should not be cited to compare to general population without context. The 7.4% (ref 27) refers to prevalence of hepatitis B among persons with HIV globally and is not much different than what the authors found in Tanzania. The discussion needs revision and several sections can be combined to summarize the prevalence of HBV infection among persons with HIV and link to suggested recommendations like screening all persons with HIV for hepatitis B and incorporating hepatitis B testing in HIV clinics as well as during antenatal care, providing hepatitis B vaccination to persons with HIV wo have not been exposed (total anti-HBc negative) and to newborns to prevent vertical transmission.

• Line 275: Older adults (aged 65 and older) do not have higher prevalence than adults aged 15-64 years. The authors need to use age groups to describe their data rather than saying older adults.

• Ref 29 is missing authors. If it is referring to the WHO guidelines, the reference needs to be updated to the more recent guidelines published in 2024. https://www.who.int/publications/i/item/9789240090903

• Discussion and associated references could also be strengthened by including the guidance of incorporating HBV screening and treatment in HIV services. https://www.who.int/publications/i/item/9789240119529

Reviewer #2: This a well written manuscripts on HIV/HBV coinfection. The authors should consider the following:

1. Describe the laboratory testing methods: for screening and confirmation of HIV and HBV infection

2. Provide any details on prevalence among pregnant women if that data was collected...if not provide this in the limitations because elimination of mother to child HBV infection is top priority.

3. Discuss infant immunization program in Tanzania to help explain the low number of vaccinated individuals in the study 200 vs 32,688. They could account for the proportion of participants who should have benefited from the global immunization program initiative from the time it was rolled out in Tanzania. How is the coverage so far?

4. In the discussion, include comparison with close neighbors such as Kenya and Uganda as part of regional data. for example, Kenya: https://pubmed.ncbi.nlm.nih.gov/39541401/

**********

what does this mean?). If published, this will include your full peer review and any attached files.

If you choose “no”, your identity will remain anonymous but your review may still be made public.

**Do you want your identity to be public for this peer review?**  For information about this choice, including consent withdrawal, please see our Privacy Policy

Reviewer #1: No

Reviewer #2: No

**********

[NOTE: If reviewer comments were submitted as an attachment file, they will be attached to this email and accessible via the submission site. Please log into your account, locate the manuscript record, and check for the action link "View Attachments". If this link does not appear, there are no attachment files.]

To ensure your figures meet our technical requirements, please review our figure guidelines: https://journals.plos.org/plosone/s/figures

You may also use PLOS’s free figure tool, NAAS, to help you prepare publication quality figures: https://journals.plos.org/plosone/s/figures#loc-tools-for-figure-preparation.

NAAS will assess whether your figures meet our technical requirements by comparing each figure against our figure specifications.

# Author comment: Author response to Decision Letter 1

**DOI:** 10.1371/journal.pone.0358242.r002

2 May 2026

Dear Sir/Madam,

Refer to the heading above, kindly find the below responses from the comments we received on our manuscript submit to your esteemed journal.

Response to reviewer 1

1. For the results, suggest first starting by describing the participant characteristics before presenting the HIV/HBV infection prevalence. Usually, results start with a general description of the study population before presenting the main outcomes.

Response: Thank you for this feedback. We accept this recommendation. We have reported participants' characteristics before presenting HIV/HBV coinfection prevalence Line 186 to 209.

2. Lines 182-184: the sentence is unclear and there is no mention of the age groups with highest HIV/HBV co-infection. It is unclear which age groups refer to the adult population, and which age groups refer to the younger population. In addition, adults are usually defined as people aged 18 and older which does not seem to be the definition that the authors are using. Recommend clearly including the age groups (25-64 years, 15-24 years, 65-80 years) that are being referred to in all sentences of this manuscript instead of mentioning adults, young and elderly. The use of the word “elderly” is not recommended, a better term is older adults.

Response: Thank you for this observation. We have incorporated clearly the age groups as per your recommendations Line 193 to 195.

3. Tables 2 and 3: For the analysis of healthcare risk factors, it is recommended to create separate variables because risks are different: receipt of blood transfusion; history of dialysis; receipt of transplant; exposure to surgical procedures. By lumping all those exposures together, authors might miss an important risk factor such as dialysis and blood transfusions.

Response: Thank you for your recommendation. We have separated the variables as recommended; however, due to a significantly high imbalance of the outcomes, we are getting very wide confidence intervals in some variables, such as history of dialysis. This was the reason we combined them in the first analysis. We have added the tables with separated variables as table 1& 3.

4. Table 3: recommend keeping age as categorical variable (15-24; 25-64; 65-80) to be consistent with the presentation in Table 2. In addition, revise the text that reports on age in multivariate analysis accordingly. I suspect that the main age group impacted by coinfection would be 15-64 years as shown in table 2. This is important especially to highlight in the discussion as they are the economically productive age groups and early deaths due to coinfection would impact economic productivity in Tanzania. Of note the lower prevalence in persons aged 65-80 is expected as most of the persons coinfected would have died earlier due to complications of their infections especially liver cirrhosis and cancer which tends to present earlier among people in Africa. https://pmc.ncbi.nlm.nih.gov/articles/PMC10204139/. https://www.sciencedirect.com/science/article/pii/S2949916X24000112

Response: Thank you for your comment. We categorized the age into (15-24; 25-64; 65-80) age groups; however, due to an imbalance in the outcome of interest, we were getting very wide confidence intervals. We have added the tables with age in groups in Table 1 for your review.

5. In the discussion, the authors mention that the overall prevalence of HIV/HBV co-infection is low, but they also point out that HBV co-infection among persons living with HIV is almost 7% which is high. Given this finding, it would be more useful if the authors analyzed factors associated with HBV co-infection among persons with HIV compared to persons who do not have HIV rather than looking at factors associated with overall prevalence of coinfection in the population. Therefore, I would recommend that the authors incorporate in their bivariate and multivariate analysis, factors associated with being co-infected with HBV among people living with HIV. For programmatic planning and to inform policy makers, it would be more useful to approach it from that perspective rather than looking at overall prevalence of HIV/HBV co-infection in the population. Policy makers need more clarity on how to integrate HBV testing and treatment among persons with HIV and the analysis using HBV infection among persons with HIV would help present clear recommendations to policy makers in Tanzania.

Response: We sincerely thank the reviewer for this thoughtful and important comment. We agree that examining factors associated with HBV co-infection specifically among people living with HIV would provide valuable programmatic insights for HIV care integration and policy planning.

However, the primary objective of our study was to characterize the burden and determinants of HIV/HBV co-infection in the general population using nationally representative data, particularly because population-level evidence on HIV/HBV co-occurrence in Tanzania remains limited. While several studies have explored HBV prevalence among people living with HIV (https://onlinelibrary.wiley.com/doi/full/10.1111/jvh.13217, https://www.frontiersin.org/journals/public-health/articles/10.3389/fpubh.2025.1565621/full, https://pmc.ncbi.nlm.nih.gov/articles/PMC5989175/), there is comparatively less evidence describing the epidemiology of HIV/HBV coinfection at the population level, which was the main knowledge gap this study sought to address.

6. Discussion lines 215-216: while hepatitis B vaccine was introduced in children as part of the pentavalent vaccine in Tanzania in 2002, the hepatitis B birth dose is not available for free and is not part of the routine immunization program in Tanzania. As for adult hepatitis B vaccination, while it might be available and mainly targets healthcare workers, there is no recommendation for universal adult vaccination against hepatitis B in Tanzania and the vaccine is not free. Reference 21 that the authors cite is focused on healthcare workers and not the general population or persons with HIV. Authors need to revise this sentence to reflect the current availability of hepatitis B vaccine in Tanzania in newborns, children and adults. The lack of hepatitis B birth dose vaccine to prevent vertical transmission of HBV is not mentioned anywhere by the authors. Vertical transmission is a key risk factor for progression to chronic HBV infection. While some persons coinfected with HIV and HBV could have acquired HBV infection at birth, this cross-sectional survey cannot identify this risk.

Response: Thank you for this important clarification regarding hepatitis B vaccination policy and availability in Tanzania. I agree that the original statement was too broad and that Reference 21 primarily reflects vaccination coverage among healthcare workers rather than the general population. I have revised the discussion to better reflect the current hepatitis B vaccination context in Tanzania line 256 – 262.

7. Lines 219-221: data from this manuscript do not support those assumptions. The only risk factor in this study were age and number of sex partners. Medical procedures and cosmetic practices were not significant. In addition, for hepatitis B, the main risk factor for chronic HBV infection is vertical transmission. The risk of progression to chronic HBV infection after exposure is up to 90% in newborns and drops to 5% in persons older than 5 years. The authors never address this risk factor in their discussion which would miss a key intervention that would further prevent HBV infections including coinfections in the future.

Response: Thank you for this important observation. I agree that the original statement required clearer alignment with the findings of this study. In our analysis, the significant factors associated with HIV/HBV coinfection were age and number of sexual partners. Medical procedures and cosmetic practices were not statistically significant predictors in our dataset. The intention of the discussion was to place our findings within the broader context of existing literature, where other studies have identified additional contributors to HBV transmission, including unsafe medical procedures, cosmetic practices involving sharps, and other blood exposure risks. However, I agree that the wording in the original manuscript could be interpreted as if these factors were directly demonstrated by our data, we have revised it line 265 to 277.

8. Lines 225-227: the prevalence of hepatitis B along persons with HIV was almost 7% in this survey, so it does match what has been reported previously in Tanzania and is not lower than previous estimates. I think authors are confusing overall prevalence of coinfection in the general population with HBV coinfection among persons with HIV. Usually, all studies assess HBV coinfection among persons with HIV as it would be most relevant for policy decisions. Hence, my previous recommendation to the analysis to assess factors associated with HBV coinfection among persons with HIV rather than the general population.

Response: Thank you for this observation. Indeed, we acknowledge the distinction between HBV coinfection prevalence in the general population versus among persons living with HIV. We have clarified this distinction in the manuscript to avoid confusion and to better contextualize our findings within the broader literature line 279-284.

9. Revise lines 222-227 to incorporate some of the information with the next paragraph starting at line 228 as they are related. All literature looked at HBV infection among persons with HIV.

Response: Thank you for this suggestion. We have revised and merged the previously separate paragraphs at lines 222–227 and 228 into a single cohesive paragraph as recommended line 278 to 284

10. Line 233: need to add the importance of introducing hepatitis B birth dose to prevent vertical transmission of HBV.

Response: Thank you for this important recommendation. We have added on hepatitis B birth dose as a critical intervention on HBV line 288 to 293.

11. Lines 237-239: Ref 28 refers to people who inject drugs and should not be cited to compare to general population without context. The 7.4% (ref 27) refers to prevalence of hepatitis B among persons with HIV globally and is not much different than what the authors found in Tanzania. The discussion needs revision and several sections can be combined to summarize the prevalence of HBV infection among persons with HIV and link to suggested recommendations like screening all persons with HIV for hepatitis B and incorporating hepatitis B testing in HIV clinics as well as during antenatal care, providing hepatitis B vaccination to persons with HIV wo have not been exposed (total anti-HBc negative) and to newborns to prevent vertical transmission.

Response: Thank you for this observation. We have incorporated the correct references and revised the section, line 296 to 324.

12. Line 275: Older adults (aged 65 and older) do not have higher prevalence than adults aged 15-64 years. The authors need to use age groups to describe their data rather than saying older adults.

Response: Thank you for this observation. We have revised on the conclusion section on the age group as per recommendation line 372.

13. Ref 29 is missing authors. If it is referring to the WHO guidelines, the reference needs to be updated to the more recent guidelines published in 2024. https://www.who.int/publications/i/item/9789240090903

Response: Thank you for this observation. We have updated the references accordingly line 324.

14. Discussion and associated references could also be strengthened by including the guidance of incorporating HBV screening and treatment in HIV services. https://www.who.int/publications/i/item/9789240119529

Response: Thank you for this guidance. We have added the consolidated guidance in our discussion section line 330.

Response to Reviewer 2

1. Describe the laboratory testing methods: for screening and confirmation of HIV and HBV infection

Response: Thank you for this recommendation. We have added this part on our method section line 134 to 143.

2. Provide any details on prevalence among pregnant women if that data was collected...if not provide this in the limitations because elimination of mother to child HBV infection is top priority.

Response: Thank you for this observation. This study was designed to assess HBV/HIV coinfection in the general adult population, pregnancy-specific estimates were outside the scope of this study.

3. Discuss infant immunization program in Tanzania to help explain the low number of vaccinated individuals in the study 200 vs 32,688. They could account for the proportion of participants who should have benefited from the global immunization program initiative from the time it was rolled out in Tanzania. How is the coverage so far?

Response: Thank you for this observation. We have expanded the discussion to contextualize the low adult vaccination coverage within Tanzania's immunization history, noting that HBV vaccination was introduced through the pentavalent vaccine in 2002 targeting infants only, adult vaccination remains largely restricted to healthcare workers, and less to the general population has been screened for HBV line 257 to 262 and line 333.

4. In the discussion, include comparison with close neighbors such as Kenya and Uganda as part of regional data. for example, Kenya: https://pubmed.ncbi.nlm.nih.gov/39541401/

Response: Thank you for this recommendation. We have added statistics accordingly line 287.

Attachment Submitted filename: Response to Reviewers.docx

# Peer review: Decision Letter 1

**DOI:** 10.1371/journal.pone.0358242.r003

**Authors:** Hans L. Tillmann

26 May 2026

Dear Dr. Mwakasungura,

Thank you for submitting your manuscript to PLOS ONE. After careful consideration, we feel that it has merit but does not fully meet PLOS ONE’s publication criteria as it currently stands. Therefore, we invite you to submit a revised version of the manuscript that addresses the points raised during the review process.

Please submit your revised manuscript by Jul 10 2026 11:59PM. If you will need more time than this to complete your revisions, please reply to this message or contact the journal office at plosone@plos.org. When you're ready to submit your revision, log on to https://www.editorialmanager.com/pone/ and select the 'Submissions Needing Revision' folder to locate your manuscript file.

- A letter that responds to each point raised by the academic editor and reviewer(s). You should upload this letter as a separate file labeled 'Response to Reviewers'.
- A marked-up copy of your manuscript that highlights changes made to the original version. You should upload this as a separate file labeled 'Revised Manuscript with Track Changes'.
- An unmarked version of your revised paper without tracked changes. You should upload this as a separate file labeled 'Manuscript'.

If you would like to make changes to your financial disclosure, please include your updated statement in your cover letter. Guidelines for resubmitting your figure files are available below the reviewer comments at the end of this letter.

If applicable, we recommend that you deposit your laboratory protocols in protocols.io to enhance the reproducibility of your results. Protocols.io assigns your protocol its own identifier (DOI) so that it can be cited independently in the future. For instructions see: https://journals.plos.org/plosone/s/submission-guidelines#loc-laboratory-protocols. Additionally, PLOS ONE offers an option for publishing peer-reviewed Lab Protocol articles, which describe protocols hosted on protocols.io. Read more information on sharing protocols at https://plos.org/protocols?utm_medium=editorial-email&utm_source=authorletters&utm_campaign=protocols.

As the corresponding author, your ORCID iD is verified in the submission system and will appear in the published article. PLOS supports the use of ORCID, and we encourage all coauthors to register for an ORCID iD and use it as well. Please encourage your coauthors to verify their ORCID iD within the submission system before final acceptance, as unverified ORCID iDs will not appear in the published article. *Only*  the individual author can complete the verification step; PLOS staff *cannot*  verify ORCID iDs on behalf of authors.

We look forward to receiving your revised manuscript.

Kind regards,

Hans L. Tillmann

Academic Editor

PLOS One

Journal Requirements:

If the reviewer comments include a recommendation to cite specific previously published works, please review and evaluate these publications to determine whether they are relevant and should be cited. There is no requirement to cite these works unless the editor has indicated otherwise.

Please review your reference list to ensure that it is complete and correct. If you have cited papers that have been retracted, please include the rationale for doing so in the manuscript text, or remove these references and replace them with relevant current references. Any changes to the reference list should be mentioned in the rebuttal letter that accompanies your revised manuscript. If you need to cite a retracted article, indicate the article’s retracted status in the References list and also include a citation and full reference for the retraction notice.

[Note: HTML markup is below. Please do not edit.]

Reviewers' comments:

Reviewer's Responses to Questions

**Comments to the Author**

Reviewer #1: (No Response)

Reviewer #2: All comments have been addressed

**********

2. Is the manuscript technically sound, and do the data support the conclusions?

Reviewer #1: Partly

Reviewer #2: Yes

**********

3. Has the statistical analysis been performed appropriately and rigorously?

Reviewer #1: Yes

Reviewer #2: Yes

**********

4. Have the authors made all data underlying the findings in their manuscript fully available?

The PLOS Data policy

Reviewer #1: Yes

Reviewer #2: No

**********

5. Is the manuscript presented in an intelligible fashion and written in standard English?

Reviewer #1: No

Reviewer #2: Yes

**********

Reviewer #1: Appreciate that the authors addressed most of my comments and revised the manuscript. However, there are still key areas that need to be addressed to ensure adequate presentation and interpretation of results.

1- Table 2: weighted percentages for the negative and no category should also be presented rather than only showing % among positive and yes.

2- Table 3: while the confidence intervals might be large if age is presented as a categorical variable or exposure to certain medical procedures, this should not restrict the presentation of those data as it is important to analyze data by age group and to differentiate medical exposures. Based on Table 1, the sample size in each age category is more than enough to present data by age groups in Table 3. It is also inadequate to lump blood transfusion with transplant and dialysis and surgical procedures. Those categories should be separated into blood transfusion (findings inform safety of blood in the country), dialysis (high risk medical intervention for acquiring HBV and HCV), and then other medical procedures (including transplant).

3- While authors are interested in overall population level prevalence of HIV/HBV co-infection, the results would not be useful from a programmatic and public health perspective as their data cannot be used to come up with key recommendations to improve HBV screening and treatment based on HIV status. Is coinfection driven by acquiring HIV in adulthood or acquiring HBV in adulthood? It seems that the coinfection is driven by the HIV infection in adults. HBV infection probably occurred in infancy and childhood. However, authors did not look at that to present key drivers of coinfection. Analyzing data looking at HBV coinfection among persons with HIV compared to those who do not have HIV has not been done at the population level in Tanzania and none of the papers cited used large scale nationally representative surveys like THIS. I would highly recommend the authors to reconsider that comment as it would not preclude them from presenting overall prevalence in the population as well. Hence, adding this analysis would help guide national viral hepatitis and HIV policies in Tanzania.

4- I would recommend revising the discussion to compile common information together and avoid redundancy. For example, lines 241-250 discuss HIV/HBV coinfection prevalence and similarly lines 259-269 discuss that same point. The ideas in those paragraphs can be combined and summarized in one paragraph. Lines 251-258 mix different ideas (hepB birth dose with integration of HBV in HIV services) and the data presented in this manuscript do not show how incorporating HBV screening and treatment in HIV clinics is useful without showing the difference in prevalence at the population level of HBV infection among persons with HIV compared to HBV infection among persons who do not have HIV.

5- The authors did not highlight that the main risk of HBV infection in high prevalence settings is perinatal and early childhood transmission. This is important to bring up as most people most likely acquired HBV infection in childhood due to MTCT and later acquired HIV infection through sexual transmission. The risk of progression to chronic HBV infection is highest among newborns and children aged <5 years. Only 5% of adults exposed to HBV progress to chronic HBV infection while up to 90% of newborns exposed to HBV progress to chronic HBV infection.

6- Reference list could be improved by removing ref 32 and 34 which are redundant and are an old guidance. This is the updated WHO guidance: https://www.who.int/publications/i/item/9789240090903

Ref 35 needs an author.

7- Recommend authors proofread their manuscript for grammatical errors before submitting. Example lines 50-52 are incoherent. Data is usually a plural term as it contains more than one piece of information (line 142: data were cleaned rather than data was cleaned). Lines 183-185: do not need to say age groups of the population (revise to among persons aged 25-64 or among persons 25-64 years of age etc.). lines 265-269.

Reviewer #2: (No Response)

**********

what does this mean?). If published, this will include your full peer review and any attached files.

If you choose “no”, your identity will remain anonymous but your review may still be made public.

**Do you want your identity to be public for this peer review?**  For information about this choice, including consent withdrawal, please see our Privacy Policy

Reviewer #1: No

Reviewer #2: No

**********

[NOTE: If reviewer comments were submitted as an attachment file, they will be attached to this email and accessible via the submission site. Please log into your account, locate the manuscript record, and check for the action link "View Attachments". If this link does not appear, there are no attachment files.]

To ensure your figures meet our technical requirements, please review our figure guidelines: https://journals.plos.org/plosone/s/figures

You may also use PLOS’s free figure tool, NAAS, to help you prepare publication quality figures: https://journals.plos.org/plosone/s/figures#loc-tools-for-figure-preparation.

NAAS will assess whether your figures meet our technical requirements by comparing each figure against our figure specifications.

# Author comment: Author response to Decision Letter 2

**DOI:** 10.1371/journal.pone.0358242.r004

10 Jul 2026

Dear Sir/Madam,

Refer to the heading above, kindly find the below responses from the comments we received on our manuscript submit to your esteemed journal.

Response to reviewer 1

1. Table 2: weighted percentages for the negative and no category should also be presented rather than only showing % among positive and yes.

Response: Thank you for this feedback. We accept this recommendation. We have added the percentages accordingly Table 2, Line 229-230.

2. Table 3: while the confidence intervals might be large if age is presented as a categorical variable or exposure to certain medical procedures, this should not restrict the presentation of those data as it is important to analyze data by age group and to differentiate medical exposures. Based on Table 1, the sample size in each age category is more than enough to present data by age groups in Table 3. It is also inadequate to lump blood transfusion with transplant and dialysis and surgical procedures. Those categories should be separated into blood transfusion (findings inform safety of blood in the country), dialysis (high risk medical intervention for acquiring HBV and HCV), and then other medical procedures (including transplant).

Response: Thank you for your insightful feedback, we have incorporated the suggestion to refine the work accordingly, Line 214 and 241.

3. While authors are interested in overall population level prevalence of HIV/HBV co-infection, the results would not be useful from a programmatic and public health perspective as their data cannot be used to come up with key recommendations to improve HBV screening and treatment based on HIV status. Is coinfection driven by acquiring HIV in adulthood or acquiring HBV in adulthood? It seems that the coinfection is driven by the HIV infection in adults. HBV infection probably occurred in infancy and childhood. However, authors did not look at that to present key drivers of coinfection. Analyzing data looking at HBV coinfection among persons with HIV compared to those who do not have HIV has not been done at the population level in Tanzania and none of the papers cited used large scale nationally representative surveys like THIS. I would highly recommend the authors to reconsider that comment as it would not preclude them from presenting overall prevalence in the population as well. Hence, adding this analysis would help guide national viral hepatitis and HIV policies in Tanzania.

Response: Thank you for your recommendation. We have included the analysis of HIV/HBV among PLHIV in our study as a sub-analysis of the study. We have added a section in the discussion on the sub-analysis as well from line 346 – 357.

4. I would recommend revising the discussion to compile common information together and avoid redundancy. For example, lines 241-250 discuss HIV/HBV coinfection prevalence and similarly lines 259-269 discuss that same point. The ideas in those paragraphs can be combined and summarized in one paragraph. Lines 251-258 mix different ideas (hepB birth dose with integration of HBV in HIV services) and the data presented in this manuscript do not show how incorporating HBV screening and treatment in HIV clinics is useful without showing the difference in prevalence at the population level of HBV infection among persons with HIV compared to HBV infection among persons who do not have HIV.

Response: Thank you for your comment. I have removed the section that creates redundancy. On Hep B vaccine this section has been removed to discuss on the findings on HIV/HBV in PLHIV.

5. The authors did not highlight that the main risk of HBV infection in high prevalence settings is perinatal and early childhood transmission. This is important to bring up as most people most likely acquired HBV infection in childhood due to MTCT and later acquired HIV infection through sexual transmission. The risk of progression to chronic HBV infection is highest among newborns and children aged <5 years. Only 5% of adults exposed to HBV progress to chronic HBV infection while up to 90% of newborns exposed to HBV progress to chronic HBV infection.

Response: We sincerely thank the reviewer for this thoughtful and important comment. We have explained this in the discussion Line 351-357

6. Reference list could be improved by removing ref 32 and 34 which are redundant and are an old guidance. This is the updated WHO guidance: https://www.who.int/publications/i/item/9789240090903 Ref 35 needs an author.

Response: Thank you for highlighting this duplicate referencing, I have removed the duplicate and updated the current guideline of 2024 accordingly, Line 306 - 308.

7. Recommend authors proofread their manuscript for grammatical errors before submitting. Example lines 50-52 are incoherent. Data is usually a plural term as it contains more than one piece of information (line 142: data were cleaned rather than data was cleaned). Lines 183-185: do not need to say age groups of the population (revise to among persons aged 25-64 or among persons 25-64 years of age etc.). lines 265-269.

Response: Thank you for your feedback I have rectified the grammar on the areas you have highlighted and proof read the whole document.

Sincerely,

Felistar Mwakasungura, MD, MSc.

Attachment Submitted filename: Response to Reviewers....docx

# Peer review: Decision Letter 2

**DOI:** 10.1371/journal.pone.0358242.r005

**Authors:** Hans L. Tillmann

29 Jul 2026

Dear Dr. Mwakasungura,

Thank you for submitting your manuscript to PLOS ONE. After careful consideration, we feel that it has merit but does not fully meet PLOS ONE’s publication criteria as it currently stands. Therefore, we invite you to submit a revised version of the manuscript that addresses the points raised during the review process.

Please submit your revised manuscript by Sep 12 2026 11:59PM. If you will need more time than this to complete your revisions, please reply to this message or contact the journal office at plosone@plos.org. When you're ready to submit your revision, log on to https://www.editorialmanager.com/pone/ and select the 'Submissions Needing Revision' folder to locate your manuscript file.

- A letter that responds to each point raised by the academic editor and reviewer(s). You should upload this letter as a separate file labeled 'Response to Reviewers'.
- A marked-up copy of your manuscript that highlights changes made to the original version. You should upload this as a separate file labeled 'Revised Manuscript with Track Changes'.
- An unmarked version of your revised paper without tracked changes. You should upload this as a separate file labeled 'Manuscript'.

If you would like to make changes to your financial disclosure, please include your updated statement in your cover letter. Guidelines for resubmitting your figure files are available below the reviewer comments at the end of this letter.

If applicable, we recommend that you deposit your laboratory protocols in protocols.io to enhance the reproducibility of your results. Protocols.io assigns your protocol its own identifier (DOI) so that it can be cited independently in the future. For instructions see: https://journals.plos.org/plosone/s/submission-guidelines#loc-laboratory-protocols. Additionally, PLOS ONE offers an option for publishing peer-reviewed Lab Protocol articles, which describe protocols hosted on protocols.io. Read more information on sharing protocols at https://plos.org/protocols?utm_medium=editorial-email&utm_source=authorletters&utm_campaign=protocols.

As the corresponding author, your ORCID iD is verified in the submission system and will appear in the published article. PLOS supports the use of ORCID, and we encourage all coauthors to register for an ORCID iD and use it as well. Please encourage your coauthors to verify their ORCID iD within the submission system before final acceptance, as unverified ORCID iDs will not appear in the published article. *Only*  the individual author can complete the verification step; PLOS staff *cannot*  verify ORCID iDs on behalf of authors.

We look forward to receiving your revised manuscript.

Kind regards,

Hans L. Tillmann

Academic Editor

PLOS One

Journal Requirements:

If the reviewer comments include a recommendation to cite specific previously published works, please review and evaluate these publications to determine whether they are relevant and should be cited. There is no requirement to cite these works unless the editor has indicated otherwise.

[Note: HTML markup is below. Please do not edit.]

Reviewers' comments:

Reviewer's Responses to Questions

**Comments to the Author**

Reviewer #1: All comments have been addressed

**********

2. Is the manuscript technically sound, and do the data support the conclusions?

Reviewer #1: Partly

**********

3. Has the statistical analysis been performed appropriately and rigorously?

Reviewer #1: I Don't Know

**********

4. Have the authors made all data underlying the findings in their manuscript fully available?

The PLOS Data policy

Reviewer #1: Yes

**********

5. Is the manuscript presented in an intelligible fashion and written in standard English?

Reviewer #1: No

**********

Reviewer #1: Thank you for addressing and responding to my comments. Some suggestions to improve the manuscript.

- Table 2: the negative category needs to also include weighted 95% confidence intervals. Or a better presentation would be to just present the positive/yes along with total denominator for each variable:

o Example:

Characteristics Number/Total tested Weighted % (95% CI)

HIV positive 1832/ 33263 4.38 (4.07-4.70)

Hepatitis B positive 1187/33263 3.53 (3.26-3.82)

Hepatitis B among PLWHIV 106/1832 6.22 (4.89-7.78)

HIV/HBV coinfection 106/33263 0.27 (0.21-0.34)

- Line 202-209: sentences need to be revised to match the results in Table 3. There is no 1.02 in Table 3. Looks like persons aged 25-64 years had higher odds of HIV/HBV coinfection than those aged 15-24 years. Same for other numbers presented in the text which do not match numbers in Table 3.

- Discussion needs to be improved by focusing on one idea per paragraph. For example, lines 254-261 and 287-298 cover multiple topics which confuses the reader.

- Manuscript needs to be proofread for clarity and English and grammar. All acronyms are defined on first use and lines 183-186 are still unclear.

**********

what does this mean?). If published, this will include your full peer review and any attached files.

If you choose “no”, your identity will remain anonymous but your review may still be made public.

**Do you want your identity to be public for this peer review?**  For information about this choice, including consent withdrawal, please see our Privacy Policy

Reviewer #1: No

**********

[NOTE: If reviewer comments were submitted as an attachment file, they will be attached to this email and accessible via the submission site. Please log into your account, locate the manuscript record, and check for the action link "View Attachments". If this link does not appear, there are no attachment files.]

To ensure your figures meet our technical requirements, please review our figure guidelines: https://journals.plos.org/plosone/s/figures

You may also use PLOS’s free figure tool, NAAS, to help you prepare publication quality figures: https://journals.plos.org/plosone/s/figures#loc-tools-for-figure-preparation.

NAAS will assess whether your figures meet our technical requirements by comparing each figure against our figure specifications.

# Author comment: Author response to Decision Letter 3

**DOI:** 10.1371/journal.pone.0358242.r006

4 Aug 2026

Dear Sir/Madam,

Refer to the heading above, kindly find the below responses from the comments we received on our manuscript submit to your esteemed journal.

Response to reviewer 1

1. Table 2: the negative category needs to also include weighted 95% confidence intervals. Or a better presentation would be to just present the positive/yes along with total denominator for each variable:

Example:

Characteristics Number/Total tested Weighted % (95% CI)

HIV positive 1832/ 33263 4.38 (4.07-4.70)

Hepatitis B positive 1187/33263 3.53 (3.26-3.82)

Hepatitis B among PLWHIV 106/1832 6.22 (4.89-7.78)

HIV/HBV coinfection 106/33263 0.27 (0.21-0.34)

Response: Thank you for this feedback. We accept this recommendation and updated table 2 accordingly.

2. Line 202-209: sentences need to be revised to match the results in Table 3. There is no 1.02 in Table 3. Looks like persons aged 25-64 years had higher odds of HIV/HBV coinfection than those aged 15-24 years. Same for other numbers presented in the text which do not match numbers in Table 3.

Response: Thank you for your insightful feedback, we have incorporated the suggestion to refine the work accordingly, Line 238 and 246.

3. Discussion needs to be improved by focusing on one idea per paragraph. For example, lines 254-261 and 287-298 cover multiple topics which confuses the reader.

Response: Thank you for your recommendation. We have separated the paragraphs for the reader to get more clarity.

4. Manuscript needs to be proofread for clarity and English and grammar. All acronyms are defined on first use and lines 183-186 are still unclear.

Response: Thank you for your comment. We have updated the section Line 188 to Line 192. We have also proofread to check for grammar.

Attachment Submitted filename: Response_To_Reviewers..._auresp_3.docx

# Peer review: Decision Letter 3

**DOI:** 10.1371/journal.pone.0358242.r007

**Authors:** Hans L. Tillmann

30 Aug 2026

HIV and Hepatitis B co-infection in Tanzania: analysis of the 2022-2023 Tanzania HIV Impact Survey.

PONE-D-26-12082R3

Dear Dr. Mwakasungura,

Thank you for following through on the requested evisions. We’re pleased to inform you that your manuscript has been judged scientifically suitable for publication and will be formally accepted for publication once it meets all outstanding technical requirements.

But please correct two minor things when proofreading [or earlier.

1.) in table 4 at section :Received Hepatitis B vaccine where it say 9 (1.00) you likely meant to say 9 (100)

2.) Lines300-302, the reviewer correctly suggests revising for accuracy as these percentages do not reflect hepatitis B chronicity rates but risk of progression to chronic HBV infection based on age of acquisition of HBV infection. “ ….estimated risk of progression to chronic HBV infection are 90% for infections acquired perinatally, 30-60% for early childhood infections and under 10% for HBV infection acquitted in adulthood. “

Thus instead of

"Importantly, in high-endemicity settings like Tanzania, .estimated risk of progression to chronic HBV infection are 90% for infections acquired perinatally, 30-60% for early childhood infections and under 10% for HBV infection acquitted in adulthood falling to under 10% in adulthood [39]"

state

"Importantly, in high-endemicity settings like Tanzania, chronic HBsAg positivity predominantly300

reflects early-life acquisition with estimated chronicity rates of 90% perinatally and 30–60% in301

early childhood, falling to under 10% in adulthood [39}"

Within one week, you’ll receive an e-mail detailing the required amendments. When these have been addressed, you’ll receive a formal acceptance letter and your manuscript will be scheduled for publication.

An invoice will be generated when your article is formally accepted. Please note, if your institution has a publishing partnership with PLOS and your article meets the relevant criteria, all or part of your publication costs will be covered. Please make sure your user information is up-to-date by logging into Editorial Manager at Editorial Manager® and clicking the ‘Update My Information' link at the top of the page. For questions related to billing, please contact billing support.

If your institution or institutions have a press office, please notify them about your upcoming paper to help maximize its impact. If they’ll be preparing press materials, please inform our press team as soon as possible -- no later than 48 hours after receiving the formal acceptance. Your manuscript will remain under strict press embargo until 2 pm Eastern Time on the date of publication. For more information, please contact onepress@plos.org.

Kind regards,

Hans L. Tillmann

Academic Editor

PLOS One

Additional Editor Comments (optional):

Reviewers' comments:

Reviewer's Responses to Questions

**Comments to the Author**

Reviewer #1: All comments have been addressed

**********

2. Is the manuscript technically sound, and do the data support the conclusions?

Reviewer #1: Yes

**********

3. Has the statistical analysis been performed appropriately and rigorously?

Reviewer #1: I Don't Know

**********

4. Have the authors made all data underlying the findings in their manuscript fully available?

The PLOS Data policy

Reviewer #1: Yes

**********

5. Is the manuscript presented in an intelligible fashion and written in standard English?

Reviewer #1: Yes

**********

Reviewer #1: Thank you for addressing and responding to my comments. Minor suggestions below for consideration.

- Table 4: I think there might be a typo error under received hepatitis B vaccine. The % should be 100% among the 9 who were vaccinated and did not have coinfection.

- Lines300-302: suggest revising for accuracy as these percentages do not reflect hepatitis B chronicity rates but risk of progression to chronic HBV infection based on age of acquisition of HBV infection. “ ….estimated risk of progression to chronic HBV infection are 90% for infections acquired perinatally, 30-60% for early childhood infections and under 10% for HBV infection acquitted in adulthood. “

**********

what does this mean?). If published, this will include your full peer review and any attached files.

If you choose “no”, your identity will remain anonymous but your review may still be made public.

**Do you want your identity to be public for this peer review?**  For information about this choice, including consent withdrawal, please see our Privacy Policy

Reviewer #1: No

**********

# Editor report: Acceptance letter

**DOI:** 10.1371/journal.pone.0358242.r008

**Authors:** Hans L. Tillmann

PONE-D-26-12082R3

PLOS One

Dear Dr. Mwakasungura,

I'm pleased to inform you that your manuscript has been deemed suitable for publication in PLOS One. Congratulations! Your manuscript is now being handed over to our production team.

At this stage, our production department will prepare your paper for publication. This includes ensuring the following:

* All references, tables, and figures are properly cited

* All relevant supporting information is included in the manuscript submission,

* There are no issues that prevent the paper from being properly typeset

You will receive further instructions from the production team, including instructions on how to review your proof when it is ready. Please keep in mind that we are working through a large volume of accepted articles, so please give us a few days to review your paper and let you know the next and final steps.

Lastly, if your institution or institutions have a press office, please let them know about your upcoming paper now to help maximize its impact. If they'll be preparing press materials, please inform our press team within the next 48 hours. Your manuscript will remain under strict press embargo until 2 pm Eastern Time on the date of publication. For more information, please contact onepress@plos.org.

You will receive an invoice from PLOS for your publication fee after your manuscript has reached the completed accept phase. If you receive an email requesting payment before acceptance or for any other service, this may be a phishing scheme. Learn how to identify phishing emails and protect your accounts at https://explore.plos.org/phishing.

If we can help with anything else, please email us at customercare@plos.org.

Thank you for submitting your work to PLOS One and supporting open access.

Kind regards,

PLOS One Editorial Office Staff

on behalf of

Dr. Hans L. Tillmann

Academic Editor

PLOS One
